# Exploring the potential of predicted miRNAs on the genes involved in the expansion of hematopoietic stem cells

**DOI:** 10.1038/s41598-024-66614-9

**Published:** 2024-07-05

**Authors:** Mohammad Elahimanesh, Nafiseh Shokri, Ronak Shabani, Maryam Rahimi, Mohammad Najafi

**Affiliations:** 1 Clinical Biochemistry Department, Faculty of Medical Sciences, Iran University of Medical Sciences, Tehran, Iran; 2 Clinical Biochemistry Department, Faculty of Medicine, Shahid Beheshti University of Medical Sciences, Tehran, Iran; 3 Anatomy Department, Faculty of Medical Sciences, Iran University of Medical Sciences, Tehran, Iran; 4 Shahid Akbarabadi Clinical Research Development Unit (ShACRDU), School of Medicine, Iran University of Medical Sciences, Tehran, Iran; 5 Clinical Biochemistry Department, Faculty of Medical Sciences, Microbial Biotechnology Research Center, Iran University of Medical Sciences, Tehran, Iran

**Keywords:** Hematopoietic stem cells, Cord blood, Proliferation, Differentiation, Gene network, miRNA, Regulatory networks, Haematopoietic stem cells

## Abstract

A major challenge in therapeutic approaches applying hematopoietic stem cells (HSCs) is the cell quantity. The primary objective of this study was to predict the miRNAs and anti-miRNAs using bioinformatics tools and investigate their effects on the expression levels of key genes predicted in the improvement of proliferation, and the inhibition of differentiation in HSCs isolated from Human umbilical cord blood (HUCB). A network including genes related to the differentiation and proliferation stages of HSCs was constructed by enriching data of text (PubMed) and StemChecker server with KEGG signaling pathways, and was improved using GEO datasets. Bioinformatics tools predicted a profile from miRNAs containing miR-20a-5p, miR-423-5p, and chimeric anti-miRNA constructed from 5′-miR-340/3′-miR-524 for the high-score genes (RB1, SMAD4, STAT1, CALML4, GNG13, and CDKN1A/CDKN1B genes) in the network. The miRNAs and anti-miRNA were transferred into HSCs using polyethylenimine (PEI). The gene expression levels were estimated using the RT-qPCR technique in the PEI + (miRNA/anti-miRNA)-contained cell groups (n = 6). Furthermore, CD markers (90, 16, and 45) were evaluated using flow cytometry. Strong relationships were found between the high-score genes, miRNAs, and chimeric anti-miRNA. The RB1, SMAD4, and STAT1 gene expression levels were decreased by miR-20a-5p (*P* < 0.05). Additionally, the anti-miRNA increased the gene expression level of GNG13 (*P* < 0.05), whereas the miR-423-5p decreased the CDKN1A gene expression level (*P* < 0.01). The cellular count also increased significantly (*P* < 0.05) but the CD45 differentiation marker did not change in the cell groups. The study revealed the predicted miRNA/anti-miRNA profile expands HSCs isolated from HUCB. While miR-20a-5p suppressed the RB1, SMAD4, and STAT1 genes involved in cellular differentiation, the anti-miRNA promoted the GNG13 gene related to the proliferation process. Notably, the mixed miRNA/anti-miRNA group exhibited the highest cellular expansion. This approach could hold promise for enhancing the cell quantity in HSC therapy.

## Introduction

It is well known that stem cells can proliferate and give rise to new differentiated cells. Hematopoietic stem cells (HSCs) are a specific class that preserves blood cell balance and are found in various sources, such as umbilical cord blood, bone marrow, and peripheral blood^1^. These cells can differentiate into other blood cells and may be used in the treatment of various blood disorders^2^. However, obtaining an applicable count of cells before they differentiate is a significant challenge for therapeutic goals^3^.

Some studies have focused on the gene profiles and signaling pathways involved in the differentiation and proliferation of HSCs^4^. A suitable population of HSCs may be produced by identifying the factors and using complementary laboratory-based therapies that control the cell cycle and differentiation processes in HSCs. According to some reports, the regulation of these processes in HSCs is greatly related to signaling pathways including WNT, NOTCH, TGFB, FGF, RAS, and JACK-STAT^5,6^. Furthermore, some genes such as Bmi-1, Nanog, Oct4, Sox2, HOXB4, and Ezh2 are reported as master regulators of differentiation and proliferation processes in the HSCs^7,8^.

By employing bioinformatics tools to analyze high-throughput data, the gene profiles might be used to find the signaling pathways involved in various cellular processes and be enriched to understand the gene functions and their regulatory factors, including miRNAs^9,10^. It is well known that the function of miRNAs may be controlled by other molecules, such as anti-miRNAs, resulting in modification of the expression of target genes^11^. Thus, a proper design for the simultaneous use of miRNAs and anti-miRNAs can affect the expression levels of genes involved in the proliferation and differentiation of HSCs.

The aim of this study was to predict the genes, miRNAs, and anti-miRNAs involved in the differentiation and proliferation processes of HSCs isolated from HUCB using bioinformatics tools. Then, the predicted miRNA/anti-miRNA profile was studied on the high-score gene expression levels to improve the expansion of HSCs.

## Materials and methods

### Gene and miRNA prediction

#### Data mining

**Figure 1:**
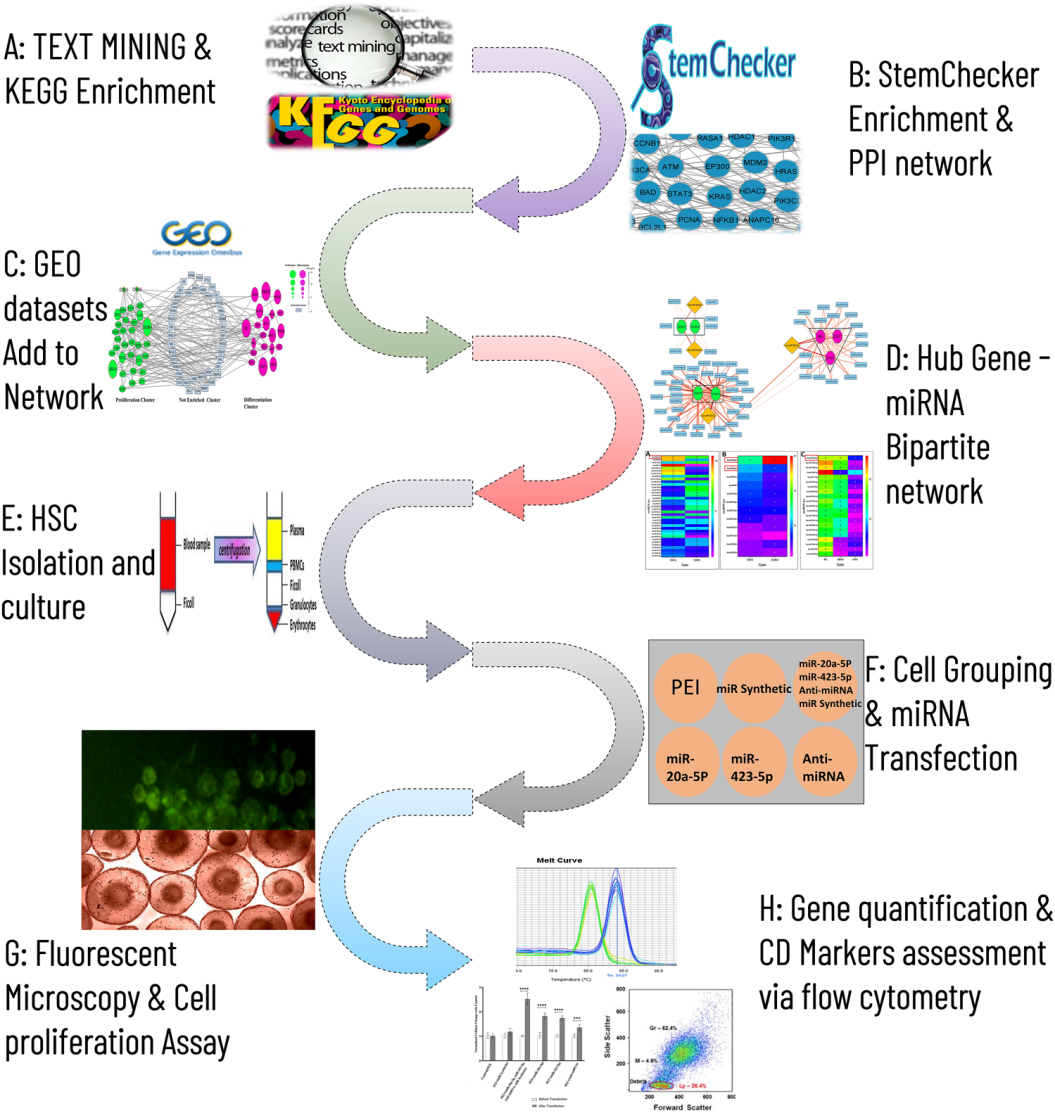
The study flowchart.

The data were collected from publications (PubMed, 2018–2024) with the keywords containing ((“Hematopoietic stem cells” OR “Blood stem cells” OR “Hematopoiesis” OR “Hematopoietic cell development” OR “Hematopoietic cell replication” OR “Hematopoietic stem cell self-renewal” OR “Hematopoietic stem cell differentiation” OR “Hematopoietic stem cell expansion” OR “Hematopoietic stem cell niche” OR “Hematopoietic stem cell transplantation”) AND (“gene regulation” OR “cell proliferation” OR “gene expression” OR “gene function” OR “hematopoietic genes” OR “gene pathways” OR “gene networks” OR “gene targets” OR “gene interactions” OR “gene therapy”)). The focus of the data collection was on identifying genes associated with their crucial roles in controlling the proliferation and differentiation processes of HSCs^3,12–28^. Furthermore, the genes were extracted and annotated from the high-throughput studies on HSCs in the StemChecker web server (http://stemchecker.sysbiolab.eu/)^29^. The StemChecker database is specialized in curating research on stem cell types. It compiles high-throughput studies such as RNA sequencing and microarrays that have identified critical genes for stem cell growth and differentiation. Then, the gene collection was enriched using the KEGG database (www.kegg.jp/kegg/kegg1.html) and the Enrichr database (https://maayanlab.cloud/Enrichr/) to identify the signaling pathways that influence the various functions of HSCs^30^ (Fig. 1A). These databases serve as a rich resource for deciphering cellular signaling pathways. By incorporating biological parameters, the databases can pinpoint pathways associated with a specific set of genes. Finally, the genes involved in the signaling pathways were the subjects to construct the gene network.

#### Gene network

A primary gene network was constructed using STRING (https://string-db.org/, interaction score > 0.9, cluster > 5 nodes) (Fig. 1B) from the genes of significant signaling pathways. Then, the gene network was transported into Cytoscape software (version 3.9.1)^31^ to visualize and improve the edges and nodes. The STRING creates a network that shows the communicational edges between genes. Furthermore, Cytoscape facilitates network manipulation and determines statistical parameters to further analyze the network during the subsequent stages.

#### Improvement of the gene network using GEO data

The gene fold data obtained from the GEO (https://www.ncbi.nlm.nih.gov/gds/?term =) datasets (GSE107497, GSE125345, and GSE179928) were used to improve the gene network (Table 1) (Fig. 1C). The datasets were analyzed with the GEO2R and R programming tools using the specialized Limma and DESeq2 packages to merge and normalize the data. The genes exhibiting significant up and low expression levels (*P*-value < 0.05 and |Log Fold Change (LFC)|> 1) in the cellular proliferation stage were compared to the differentiation process (Table 2) so that the intersection of genes were defined two sets. (1) The high and low expression genes (n = 108) predicted in the proliferation and self-renewal of HSCs. (2) The high and low expression genes (n = 103) in the differentiation stage of HSCs. The nodes on the gene network were improved using the absolute values of gene fold data and displayed the proliferation and differentiation clusters.

**Table 1:** GEO dataset.

GEO accession	Sample	PMID	Experiment type	Platform
GSE107497	19	29,701,016	Expression profiling by array	GPL17586
GSE125345	15	31,631,013	Expression profiling by high throughput sequencing	GPL16791
GSE179928	8	NA	Expression profiling by high throughput sequencing	GPL18573

**Table 2:** Genes with the significant expression levels in differentiated cells and HSCs.

GEO dataset^a^	High express genes in HSCs (n)	Low express genes in HSCs (n)	High express genes in differentiated cells (n)	Low express genes in differentiated cells (n)
GSE107497	201	159	70	152
GSE125345	2599	2758	2508	1670
GSE179928	4275	4790	3209	4500

^a^GSE107497 (GPL17586) compared the gene expression patterns of HSCs, myeloid progenitor cells, and erythroid progenitor cells with those obtained directly from umbilical cord blood. GSE125345 (GPL16791) reported the gene expression data for various types of blood cells, including long-term HSCs, short-term HSCs, multipotent progenitors, common myeloid progenitors, granulocyte and monocyte progenitors, and multilymphoid progenitors from cord blood. GSE179928 (GPL18573) performed RNA-seq analysis on two types of cord blood CD34 + cells: freshly isolated HSCs and 4-day ex-vivo cultured HSCs. Genes with a *p*-value < 0.05 and a log fold change (LogFC) > 1 were regarded as high expressed genes, while genes with a *p*-value < 0.05 and a LogFC < -1 were defined as low expressed genes.

#### Gene‑miRNA bipartite relationship

To pinpoint miRNAs that potentially regulate the high-score nodes (high-size nodes) in HSC proliferation and differentiation stages, the miRTarBase, DIANA-TarBase, and ENCORI/starBase databases were applied. Each database employs distinct algorithms and experimental data to predict miRNA-target gene interactions. The scores in each database reflect the strength of prediction or confidence of binding miRNA to the target gene. Higher scores typically indicate a more likely interaction (Fig. 1D). The final grading of the gene-miRNA bipartite was obtained from the sum of database scores and was represented as edge thickness.

### Cellular isolation and culture

The HUCB bags (25 ml, citrate, phosphate, and dextrose) containing the suitable HSC counts were evaluated and obtained by the Iranian Blood Transfusion Organization (IBTO) in conformity with the community ethical committee’s ethical guidelines (IR.IUMS.FMD.REC.1402.048). Each UCB unit was diluted 1:1 with phosphate buffered solution and transferred into a 50 ml tube containing 15 ml Ficoll Hypaque solution (Amersham Pharmacia, Piscataway, NJ; density, 1.077 g/mL). Mononuclear cells (MNCs) were obtained from the interphase layer (above the Ficoll layer) after centrifuging at 2800 rpm (25 min) (Fig. 1E). MNCs were then washed twice with DMEM F12 (10% FBS, Gibco, Thermo Fisher Scientific-US), re-suspended, and seeded in DMEM F12 media (20% FBS, 1% Pen-Strep (Sigma-Aldrich Co., St Louis, MO, USA)) in humidified conditions (37 °C, 5% CO_2_). Some studies reported that SCF, TPO and other growth factors may trigger the differentiation process^32,33^. To diminish these effects, the medium was limited with these factors and supplemented with 150 µg/ml apo-transferrin^34^, 50 µg/ml insulin, 20 units/ml Erythropoietin (EPO), 50 ng/ml growth hormone (GH)^35,36^, vitamin A and E each 50 µM, nicotinamide 10 mM,^37–39^ and non-essential amino acids (NEAA)^40^ (Gibco, cat No. 11140–035). The non-adherent cells including HSCs, were isolated during several passages^41^. In each passage, the adherent cells were discarded, and the supernatant cells were used only for the next passage.

### Experimental study

#### Cell groups

The gene fold data of GSE179928 obtained in 4-day period were used to improve the nodes on the gene network. However, to better observe the variance changes of genes and the self-renewal and differentiation CD markers that may appear due to the time-dependent cell phenotype events, the pre-interventional culture period of HSCs increased up to 6 days as the longer periods were reported in other studies^35,42^. On the sixth day, the cultured (enriched DMEM F12) cells (1 × 10^6^) were divided into six groups, including the polyethyleneimine (PEI) (25 kDa), PEI + miR Synthetic (non-functional nucleotide sequence), PEI + miR-20a-5p, PEI + miR-423-5p, PEI + anti-miRNA, and PEI + miR Synthetic + miR-20a-5p + miR-423-5p + and anti-miRNA groups (Fig. 1F).

#### Transfer of miRNA/anti-miRNA into HSCs

The anti-miRNA was designed by connecting 11 reverse complement bases of 5′miR-340-5p (MIMAT0004692: 5′-UUAUAAAGCAAUGAGACUGAUU-3′) and 11 reverse complement bases of 3′miR-524-5p (MIMAT0002849: 5′-CUACAAAGGGAAGCACUUUCUC-3′), which were separated from each other with AT nucleotide bases so that the chimeric construct might bind to both the miRNAs (Table 3). PEI (Sigma Aldrich, Cat. No. 408,727) was used to transfer the miRNA/anti-miRNA into HSCs. A solution of PEI was prepared by shaking at 37 °C (20 mg/ml DEPC water). Also, a solution of miR-20a-5p (MIMAT0000075), miR-423-5p (MIMAT0004748), anti-miRNA, and miR Synthetic were prepared and incubated at room temperature for 20 min (100 pM). Based on the cell groups, 1 µl of each miRNA/anti-miRNA solution and 1 µl of PEI solution were added into 200 µl of DEPC water and incubated at room temperature for 30 min. Then, the mixture was added to 1800 µl of additional culture medium on the sixth day^43^. After 4 h, the cells were washed with PBS buffer, re-cultured and, harvested after one day for RNA extraction.

**Table 3:** Oligonucleotide sequences used in the study.

Sequence name	Nucleotide sequence
miR-20a-5p	5′- TAAAGTGCTTATAGTGCAGGTAG -3′
miR-423-5p	5′- TGAGGGGCAGAGAGCGAGACTTT -3′
anti-miRNA	5′- TTGCTTTATAAATGAGAAAGTGCT -3′
miR Synthetic	5′- TCCCTTTGTAGCACAATCAGTCTCA -3′
FAM-probe	5′- 6-FAM CCCGAGACCCAACTGGTCACC -3′

A fluorescently labeled oligonucleotide (5′-6-FAM-CCCGAGACCCAACTGGTCACC -3′), as a green fluorescence emitter, was used to evaluate cellular delivery. Following the transfer of labeled oligonucleotide with PEI, the cells were incubated at 37 °C with 5% CO_2_ for 4 h before being washed with PBS buffer and imaged using an Olympus IX71 microscope (Fig. 1G).

#### Flow cytometry

The cell supernatant containing HSCs was centrifuged for 7 min at 1500 rpm. Then, the cells (3 × 10^4^) were gently suspended in PBS buffer and treated using the labeled antibodies against CD90, CD45, and CD16 markers (Abcam in Cambridge, United Kingdom) for 25 min at room temperature (Fig. 1H).

#### RNA extraction, cDNA synthesis and RT-qPCR

After 24 h from the intervention period (4 h), the cells were counted and total RNA was extracted using the SinaPureTM ONE kit (Sinaclon, Tehran, Iran, Cat. No. EX6051). The Yektatajhiz cDNA Synthesis Kit (Tehran, Iran, Cat. No. YT4500) was used to synthesize the cDNA. The Real-time qRT-PCR technique was carried out using YTA SYBR Green qPCR Master mix (Yektatajhiz, Cat. No. YT2551). The GAPDH gene was used to normalize the gene expression levels. The reactions for all genes were run in 15 µl volumes. To design gene primers, the Primer-BLAST (https://www.ncbi.nlm.nih.gov/tools/primer-blast/) and OligoAnalyzer servers (https://eu.idtdna.com/pages/tools/oligoanalyzer) were used (Table 4).

**Table 4:** Primer sequences.

Gene	Primer sequence
CDKN1A-Forward CDKN1A-Reverse	5′- AGGGGACAGCAGAGGAAGAC -3′ 5′- CGGCGTTTGGAGTGGTAGAA -3′
CDKN1B-Forward CDKN1B-Reverse	5′- TGAGGACACGCATTTGGTGGA -3′ 5′- GTCGGTTGCAGGTCGCTTC -3′
GNG13-Forward GNG13-Reverse	5′- AGATGAAGAAAGAGGTGGAGAGCC -3′ 5′- TCATCAGGTCGGGGTTCAGGA -3′
RB1-Forward RB1-Reverse	5′- AGGTCTGCCAACACCAACAA -3′ 5′- AGGGTTGCTTCCTTCAGCAC -3′
SMAD4-Forward SMAD4-Reverse	5′- TGCCTCACCACCAAAACGG -3′ 5′- AACACCAATACTCAGGAGCAGG -3′
STAT1-Forward STAT1-Reverse	5′- AAAGGAAGCACCAGAGCCAA -3′ 5′- GAGCCCACTATCCGAGACAC -3′
CALML4-Forward CALML4-Reverse	5′- GACGGAAATGGAGAGCTGGA -3′ 5′- TCGTGAGTTTTGACCGCAGG -3′
GAPDH-Forward GAPDH-Reversed	5′- CATGAGAAGTATGACAACAGCCT -3′ 5′- AGTCCTTCCACGATACCAAAGT -3′

### Statistical analysis

The gene expression datasets were analyzed in R software (Version 4.3.0). The Kolmogorov–Smirnov test was used to assess the parametric and non-parametric distributions. Student's t-test, ANOVA, and Tukey’s post hoc test were used to compare the cellular groups in GraphPad Prism (Version 8.4.3). The flow cytometry data was analyzed with FlowJo software (Version 10). Using the 2^−ΔΔCT^ formula, the changes in gene expression levels were estimated. *P*-values that were equal to or less than 0.05 were regarded as significant differences between the groups.

### Ethics approval and consent to participate

The project titled “Systematic and functional studies of microRNA and anti-microRNA affecting genes involved in the growth process of hematopoietic stem cells isolated from human umbilical cord blood cells” was agreed by Research Ethics Committee of School of Medicine (IUMS). A research Ethics Committee certificate was presented and attached. (Ethics Co. IR.IUMS.FMD.REC.1402.048). The study was accomplished with a waiver for acquiring informed consent due to applying the unused human umbilical cord blood (UCB) bags.

### Human and animal rights

Hematopoietic stem cells were prepared from the unused human umbilical cord blood (UCB) stored in the Iran Blood Transfusion Organization (IBTO).

## Results

The prediction and experiment studies are shown in a flowchart (Fig. 1A–H).

### RAS, PI3K-Akt, JAK-STAT, and cell cycle signaling pathways were predicted in the proliferation and differentiation processes of HSCs

The bioinformatics tools were used to analyze the gene data and to predict their roles in the cellular signaling pathways. The text mining analysis revealed 441 genes that were crucial for the differentiation and proliferation processes of HSCs. The major signaling pathways (KEGG) related to these genes included the PI3K-Akt signaling pathway (hsa04151, 73 genes), pathways in cancer (hsa05200, 66 genes), JAK-STAT signaling pathway (hsa04630, 62 genes), cell cycle (hsa04110, 59 genes), cytokine-cytokine receptor interaction (hsa04060, 59 genes), MAPK signaling pathway (hsa04010, 44 genes), Ras signaling pathway (hsa04014, 41 genes), Rap1 signaling pathway (hsa04015, 36 genes), and calcium signaling pathway (hsa04020, 27 genes). With annotating the suggested genes from the StemChecker database (Supplement 1 and 3), the four PI3K-Akt (2.36 E^-109^), RAS (1.85 E^-42^), JAK-STAT (1.43 E^-73^), and cell cycle (8.38 E^-79^) signaling pathways were more significant (Supplement 4) so that their genes were applied to generate the gene network in the STRING server (462 nodes, and 2221 edges) (Supplement 5A).

### RB1, SMAD4, STAT1, CALML4, GNG13, and CDKN1A/CDKN1B genes, as well as miR-423-5p, miR-340-5p, miR-524-5p, and miR-20a-5p were predicted to control the major signaling pathways

**Figure 2:**
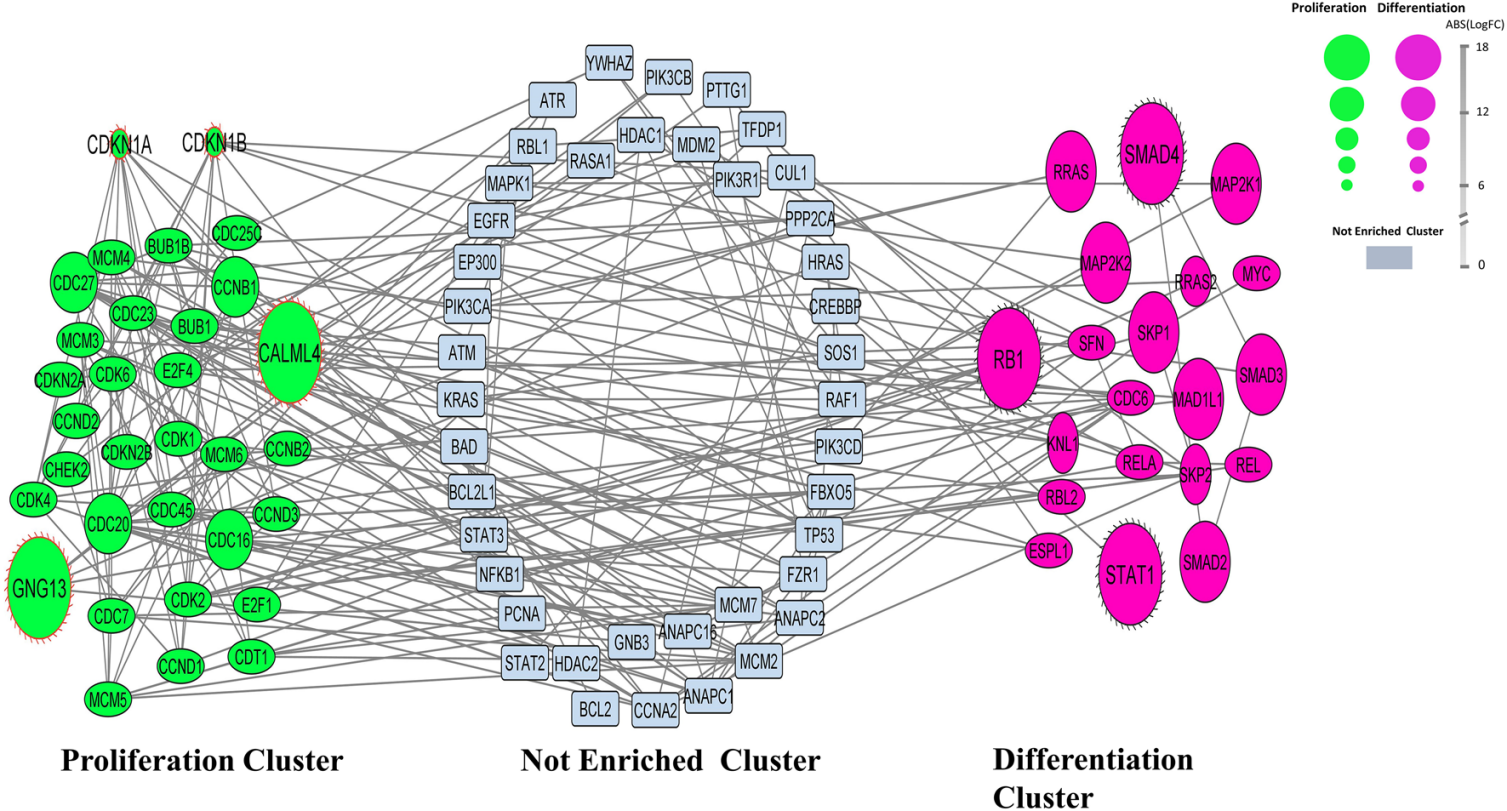
Gene network. (**A**) The green nodes represents the genes involved in the proliferation process of HSCs. (**B**) The light blue nodes show the genes involved in the growth of HSCs but were not improved using the gene expression datasets. (**C**) The pink nodes represents the genes involved in the differentiation process. The node size is indicated as the absolute value of gene fold of expression datasets.

**Figure 3:**
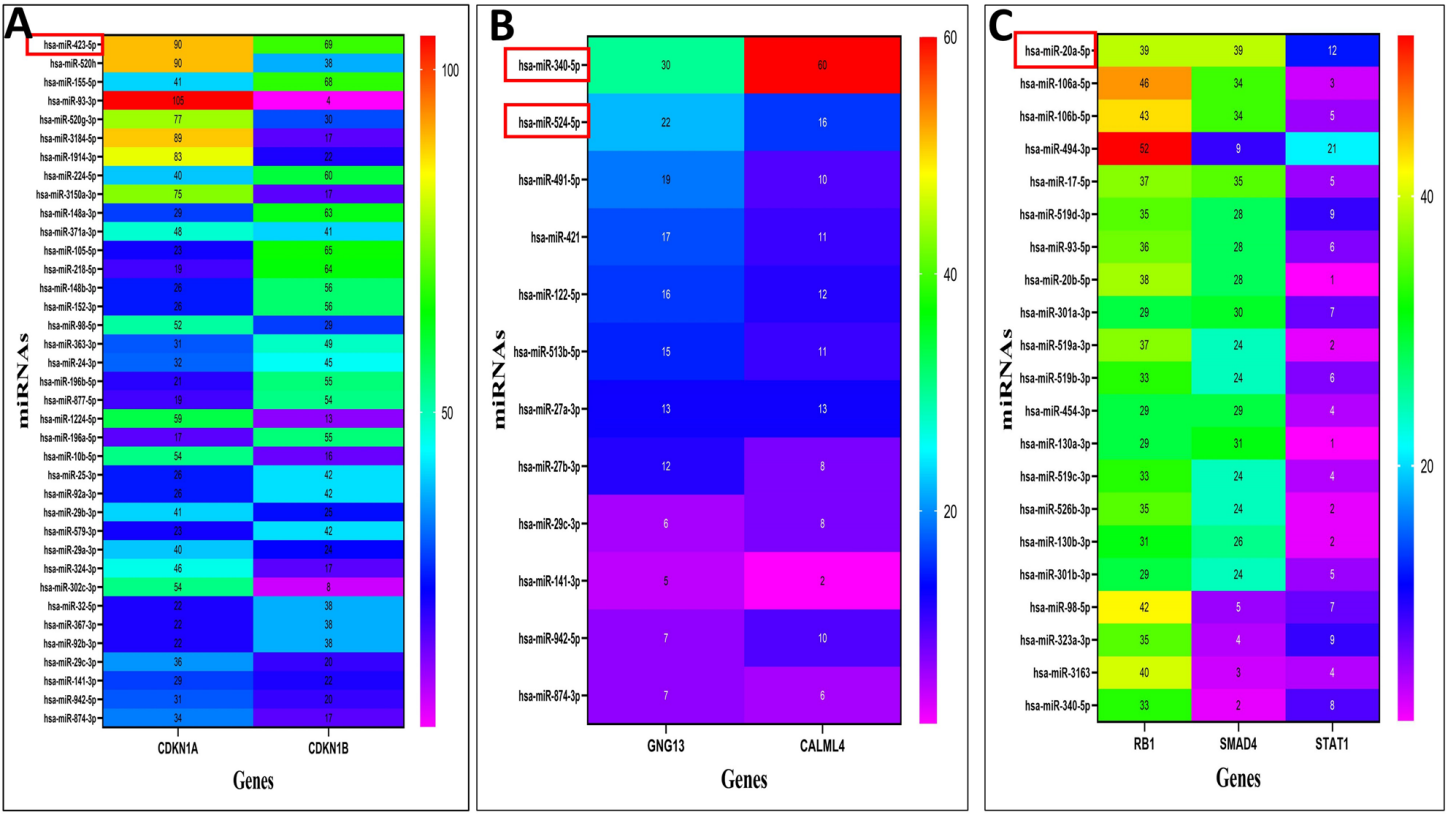
Heatmap graphs between miRNAs and target genes in the HSCs. (**A**) Top miRNAs targeting the CDKN1A and CDKN1B genes involved in the proliferation process. (**B**) Top miRNAs targeting the GNG13 and CALML4 genes related to the proliferation process. (**C**) Top miRNAs targeting the RB1, SMAD4, and STAT1 genes involved in the differentiation process.

**Figure 4:**
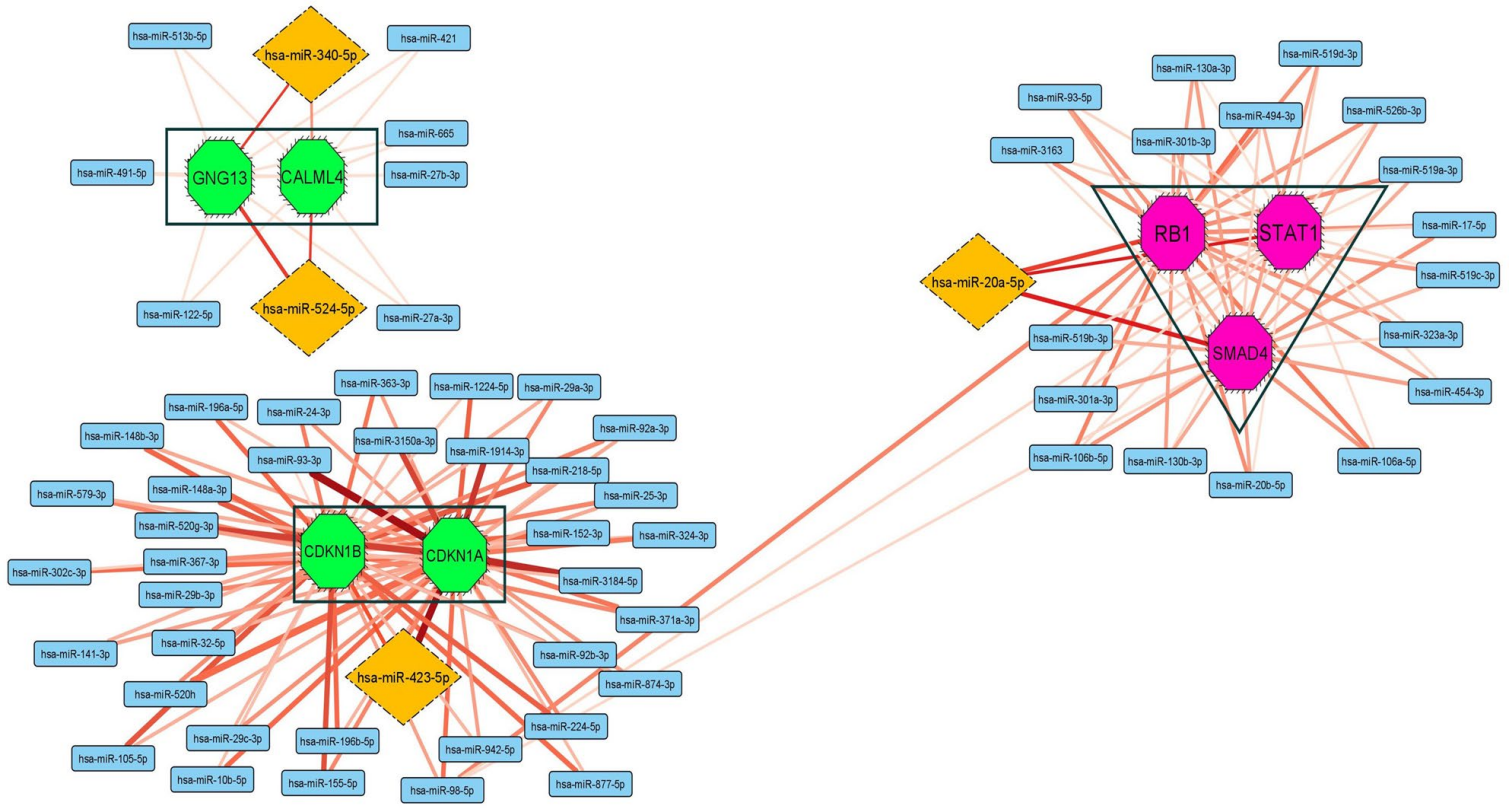
Gene-miRNA network. The multi-interactions between the miRNAs and the target genes in the proliferation (green) and differentiation (pink) processes. The edges show the total score estimated from the miRNA databases.

The roles of miRNAs were predicted on the high score genes using the gene-miRNA network. The absolute values of fold data of HSC proliferation (n = 108) and differentiation (n = 103) genes estimated from GSE datasets (Supplement 2) were annotated to the nodes (green and pink colors) to improve and categorize the gene network. Some genes of the network that were not observed in the datasets according to the gene selection criteria were shown in light blue (Fig. 2). High- and low-fold genes including RB1 (+ 12.48), SMAD4 (+ 16.08), and STAT1 (+ 13.52) in the differentiation cluster (containing pink nodes) and CALML4 (+ 18.09), GNG13 (+ 17.97), CDKN1A (− 17.74), and CDKN1B (− 17.99) in the proliferation cluster (containing green nodes) were predicted on the gene network. Figure 3 displays the miRNAs related to the predicted genes. While miR-423-5p predicted to target both the CDKN1A and CDKN1B genes, miR-340-5p and miR-524-5p targeted the CALML4 and GNG13 genes. Furthermore, miR-20a-5p targeted the RB1, SMAD4, and STAT1 genes (Fig. 4).

### The miRNA/anti-miRNA were efficiently transferred to HSCs by PEI

The cells were transfected with a FAM-ligated probe to evaluate the cell delivery. The fluorescent microscopic images showed that PEI transfers efficiently miRNA/anti-miRNA into the HSCs (Supplement 5B).

### CD16, CD90, and CD45 were estimated during the study

**Figure 5:**
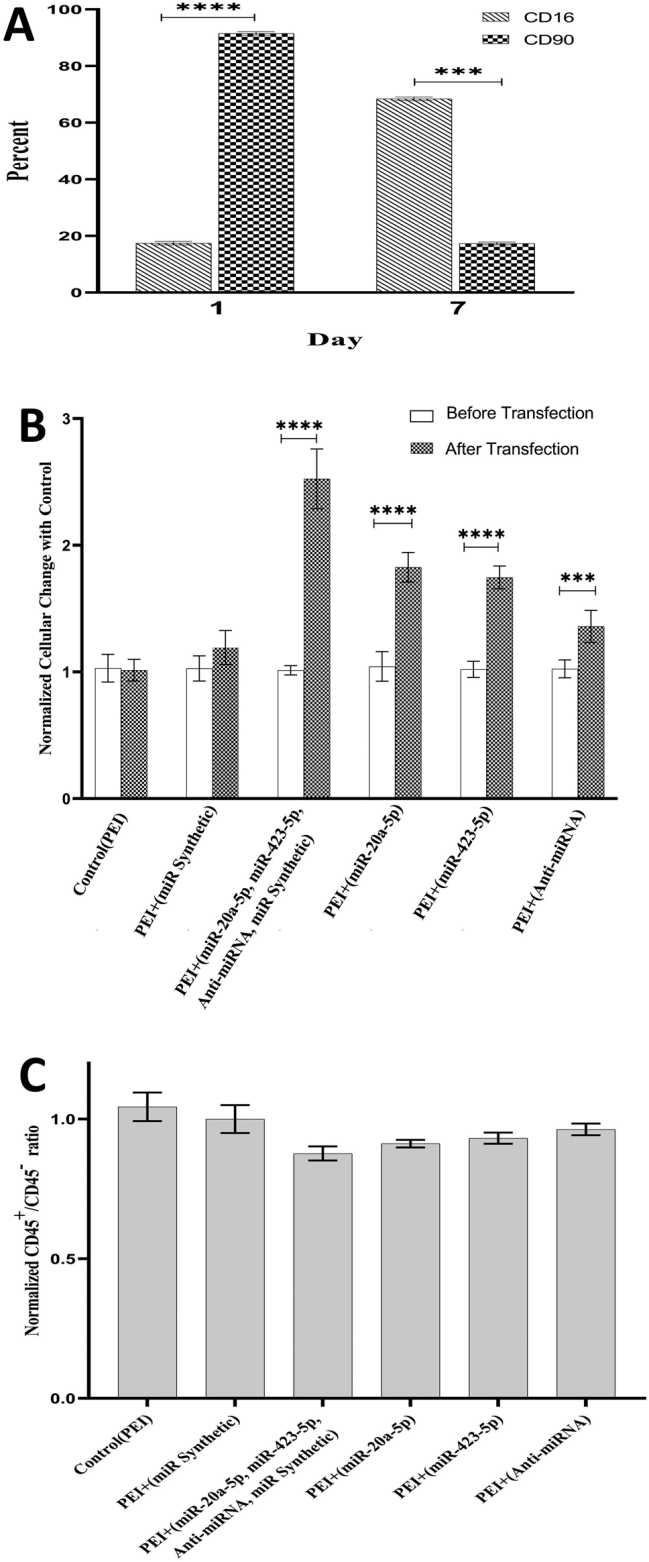
The changes of CD markers and cell count in the HSCs. (**A**) The CD90 and CD16 markers on the first and seventh days. (**B**) The cell count in the study cellular groups. (**C**) The CD45^+^/CD45^-^ ratios in the cellular groups on the seventh day.

The cells were analyzed with flow cytometry for the expression of CD90 and CD16 to determine the respective proportion of HSCs and differentiated cells on the first day after culturing the cells and on the seventh day. The CD16 and CD90 were estimated 17.9 ± 0.35% and 91.4 ± 0.65% one day after culturing the cells, respectively. However, their values changed conversely after the seventh day (CD16 31.1 ± 0.39%, CD90 17.7 ± 0.59%) (Supplement 5C) so that CD16 increased up to 1.73 times (*p* 0.035) while CD90 decreased by 5.16 times (*p* 0.0001). Also, the CD45 differentiation marker was estimated 78.6 ± 0.53% on the seventh day (Fig. 5A).

### The miRNAs and anti-miRNA changed the cell count

The cell count was estimated as the proliferation rate of HSCs. The number of cells increased significantly in the PEI + (containing miR Synthetic, miR-20a-5p, miR-423-5p, and anti-miRNA) (2.66-fold, *p* 0.0001), PEI + miR-20a-5p (1.962-fold, *p* 0.0001), PEI + miR-423-5p (1.9-fold, *p* 0.0001) and PEI + anti-miRNA (1.43-fold, P 0.0001) groups as compared to the PEI + miR Synthetic group (Fig. 5B, Supplement 5D).

### The miRNAs and anti-miRNA did not change CD45 marker

The changes of CD45 marker were compared between the cell groups as the differentiation rate of HSCs. There were no significant differences in the normalized CD45^+^/CD45^-^ ratios among the groups. However, this ratio (0.877) was lower in the PEI + (miR-20a-5p, miR-423-5p, anti-miRNA, miR Synthetic) group. In other groups including PEI + miR-20a-5p, PEI + miR-423-5p, and PEI + anti-miRNA, the ratios were 0.912, 0.931, and 0.962, respectively (Fig. 5C, Supplement 5E).

### The GNG13, CDKN1A, RB1, SMAD4, and STAT1 gene expression levels were changed using miRNAs and anti-miRNA

**Figure 6:**
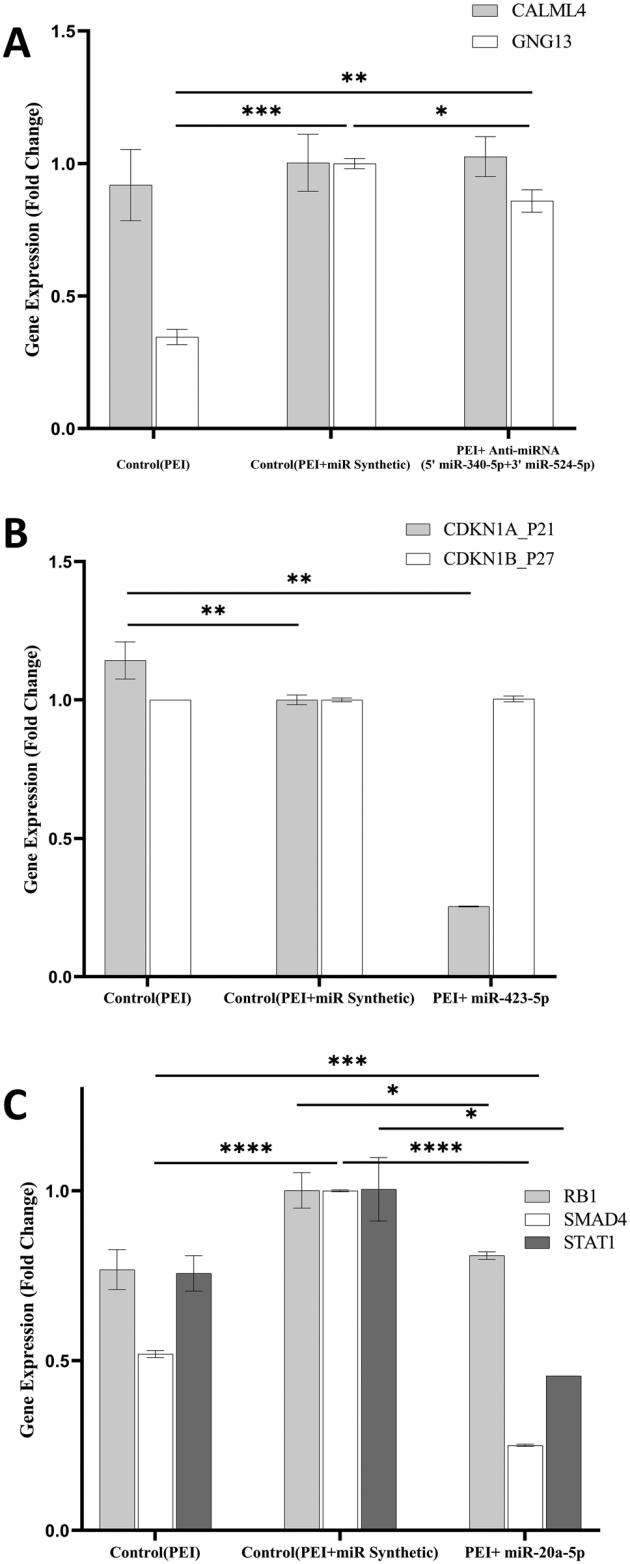
The gene expression changes in the HSCs. (**A**) The effects of anti-miRNA on the CALML4 and GNG13 genes. (**B**) The effects of miR-423-5p on the CDKN1A and CDKN1B gene expression levels. (**C**) The effects of miR-20a-5p on the RB1, SMAD4, and STAT1 gene expression levels. The data are presented as mean ± SD.

The gene expression levels were evaluated by the RT-qPCR technique. The CALML4 gene expression levels did not change significantly using anti-miRNA as compared to the PEI and PEI + miR Synthetic groups (*p* > 0.9) in the HSCs. However, the GNG13 gene expression levels increased significantly (*p* < 0.05) (Fig. 6A). The results also showed that CDKN1A gene expression levels decreased significantly in miR-423-5p/PEI-transfected cells as compared to the PEI + miR Synthetic and PEI groups (*p* 0.002 and *p* 0.001, respectively). However, the CDKN1B gene expression levels were not significantly changed in miR-423-5p/PEI-transfected cells (*p* > 0.8) (Fig. 6B). The RB1 gene expression levels decreased in the transfected cells with PEI + miR-20a-5p as compared to the transfected group with PEI + miR Synthetic (*p* 0.046). Similarly, the SMAD4 and STAT1 gene expression levels were lower in the miR-20a-5p/PEI-transfected cells (*p* 0.0001 and *p* 0.016, respectively) (Fig. 6C).

## Discussion

HSCs are used in the treatment of blood disorders, metabolic deficiencies, and immune system abnormalities^44^. The standout features of umbilical cord blood HSCs include their non-invasive and painless blood collection methods, abundant presence of primitive and progenitor cells, capacity for cellular expansion, and high tolerance for the prevention of transplant rejection. Furthermore, HSCs can be used to regenerate damaged tissues, replace lost cells, and modulate the immune system. However, the limited number of HSCs may restrict the potential of these treatments^45,46^. It is well known that the expansion and differentiation of HSCs might be related to different internal and external factors. The external factors such as the microenvironment, adhesion molecules, growth factors, cytokines, chemical compounds, and co-culturing with other cells via the internal factors like signaling pathways can help to expand and differentiate HSCs^4,13, 18, 47^. Thus, the control of cell proliferation and differentiation processes of HSCs is an interesting subject^48,49^.

In this study, the data mining related to the proliferation and differentiation processes of HSCs were improved using the GEO datasets in agreement with the previous report^50^. In the proliferation stage, the GNG13 and CALML4 genes predicted the highest expression fold values, whereas CDKN1A and CDKN1B had the lowest. In the differentiation stage, the RB1, SMAD4, and STAT1 genes revealed the highest expression fold values. Based on the bioinformatics results, it was hypothesized to downregulate the CDKN1A, CDKN1B, STAT1, SMAD4, and RB1 genes and to upregulate the CALML4 and GNG13 genes for the improvement of proliferation and the prevention of differentiation of HSCs. Since some miRNAs were reported to regulate the genes involved in the proliferation and differentiation processes in HSCs^51,52^ thus in this study, the predicted genes were suggested to be targeted by miRNAs. The miR-20a-5p proposed the strongest ability to suppress the expression of three genes (RB1, SMAD4, and STAT1) in the differentiation process, while miR-423-5p suggested the highest potential to decrease the expression of two genes (CDKN1A and CDKN1B) that can inhibit proliferation stage. An anti-miRNA was designed to increase the expression of GNG13 and CALML4 genes by inhibiting miR-340-5p and miR-524-5p.

It was an effort to separate the non-adherent cells such as HSCs and hematopoietic progenitors through successive passages, separation, and culturing of supernatant cells at each stage^41^. Since attaching antibodies to cells and subjecting them to different reagents over an extended period can affect the typical states of the HSCs^53^ thus, it is crucial to minimize the loss of hematopoietic progenitor cells in a suitable microenvironment. For this reason, in line with the objective of this study to decrease the differentiation rate of HSCs and hematopoietic precursors while promoting their proliferation, a sequential partition purification method was employed and the cellular surface markers were evaluated. CD90 is a marker commonly expressed on many cells including HSCs, mesenchymal stem cells (MSCs), and keratinocytic stem cells (KSCs)^54–58^. The results suggested that the reduction of the CD90 marker (5 folds) following the sequential cell passages might be due to the omission of some cells including adherent stem cells on the seventh day. CD16 is also found in various differentiated cells such as natural killer cells, HSCs, neutrophils, monocytes, macrophages, and certain T cells^59^. Thus, its increase may occur because of the elevated differentiation of non-adherent cells such as HSCs on the seventh day. CD45 is present in various types of differentiated blood cells, excluding mature red blood cells and platelets^60^. Moreover, the predicted gene changes were evaluated on the seventh day since the CD16 and CD45 showed the cells shed their stemness conditions and developed into differentiated cells within seven days. The study results revealed that the anti-miRNA enhance the proliferation of HSCs. The anti-miRNA increased GNG13 gene expression levels, which may explain the reason for the enhanced cell proliferation. GNG13 is reported to be expressed in many tissues and contributes as a signal transmitter^61,62^. There were also many reports on the CDKN1A and CDKN1B genes and their impacts on cellular expansion. CDKN1A can inhibit cell cycle and tumor growth^24^. The increase in CDKN1A and CDKN1B gene expression levels maintained the quiescent state of HSCs and prevented the stem cell self-renewal^63^. Moreover, the growth of hepatocellular carcinoma is promoted by miR-423 via suppressing the expression of the tumor suppressor CDKN1A^64^. In agreement with these reports, the study results showed that the decrease in the CDKN1A gene expression by miR-423-5p improves the cell proliferation. In contrast with the gene-miRNA prediction results, however, there were no significant alterations due to the effects of anti-miRNA and miRNA on the CALML4 and CDKN1B gene expression levels. However, calcium is connected to CALML and is involved in the FGF, Ras, MAPK, and Wnt signaling pathways^65^. The activation of the MAPK pathway also affected the proliferation and differentiation of stem cells by a rise in the cytosolic calcium levels^66^. Some substances such as G-CSF, SCF, EPO, IL-3, and IL-6 increased the cellular calcium concentration leading to the proliferation and differentiation of the HSCs^67^.

The prediction results also suggested that the RB1, SMAD4, and STAT1 gene expression levels were elevated in the differentiation of HSCs. The experiment results showed the RB1, SMAD4, and STAT1 gene expression levels decreased by miR-20a-5p, in line with the gene-miRNA prediction results. Compared to the control group, however, the proliferation of HSCs was enhanced, but the CD45 marker, as a differentiation marker, had no change in the cell groups. Some studies agreed with our results on the involvement of RB1 in the cell cycle and differentiation^68,69^. Furthermore, the TGF-beta signaling pathway affected cellular growth via the SMAD4 isoforms attributing an antiproliferative response^70^. SMAD4 also regenerated and expanded the ability of human HSCs^71^. miR-130a decreased the sensitivity of TGF-β1-induced growth inhibition in a granulocytic cell line by suppressing the production of SMAD4^72^. When differentiation-inducing cytokines bind to receptors on the surface of cells, they phosphorylate the STAT signaling molecule. The phosphorylated STATs (STAT1, STAT3, STAT5) combine to form dimers, which then move into the nucleus to regulate gene transcription. During all-trans retinoic acid (ATRA)-induced differentiation, STAT1 stops the cell cycle by regulating cyclins, p27, and c-Myc^73,74^. Megakaryopoiesis is significantly influenced by STAT1^75^. On the other hand, miRNAs were able to change the gene expression levels. Some studies indicated a decrease in RB1 gene expression by miR-20a-5p^76,77^. The miR-20a-5p also suppressed the expression of SMAD4 in colorectal cancer cells^78^. The levels of miR-20a-5p may regulate the ability of human endometrium-derived mesenchymal stem cells (hEMSCs) to differentiate into cardiac cells, possibly by controlling the levels of SMAD4^78^. The study results showed that in addition to miR-20a-5p, the combinational miRNA/anti-miRNA use elevates the proliferation process while preventing differentiation in HSCs.

## Conclusion

The bioinformatics results predicted the list of genes and miRNAs related to the proliferation and differentiation stages of HSCs. Based on these data, some high-score genes and miRNAs were experimented in the HSCs. The study results confirmed that miR-423-5p and anti-miRNA promote the proliferation of HSCs by influencing the CDKN1A and GNG13 gene expression levels. Also, miR-20a-5p prevented the differentiation process in HSCs by suppressing the RB1, SMAD4, and STAT1 gene expression levels. Additionally, the combinational miR-423-5p/miR-20a-5p/anti-miRNA use caused a considerable increase in the proliferation process of HSCs.

Further studies are needed to understand the mechanism roles of miRNAs on the expression levels of suggested genes and to evaluate their protein values related to down- and upstream genes to gain knowledge of the signaling pathways involved in the proliferation and differentiation processes in the HSCs.

### Supplementary Information

Supplementary Information 1.

Supplementary Information 2.

Supplementary Information 3.

Supplementary Information 4.

Supplementary Information 5.

## Data Availability

The datasets analyzed during the current study are available in the GEO repository (https://www.ncbi.nlm.nih.gov/GEO). GSE107497, GSE125345, and GSE179928. All data are available in the main text or the supplementary materials.
