# Supplementary material for: Exploring the potential of predicted miRNAs on the genes involved in the expansion of hematopoietic stem cells
Source: Sci Rep. 2024 Jul 5;14:15551. doi: 10.1038/s41598-024-66614-9 (PMC11226654; doi:10.1038/s41598-024-66614-9)
Supplement: Supplementary file 5 — Supplementary Information 5. [file 41598_2024_66614_MOESM5_ESM.pdf]

Supplement 5: The gene network, miRNA transfection, CD estimation and cell count in HSCs.

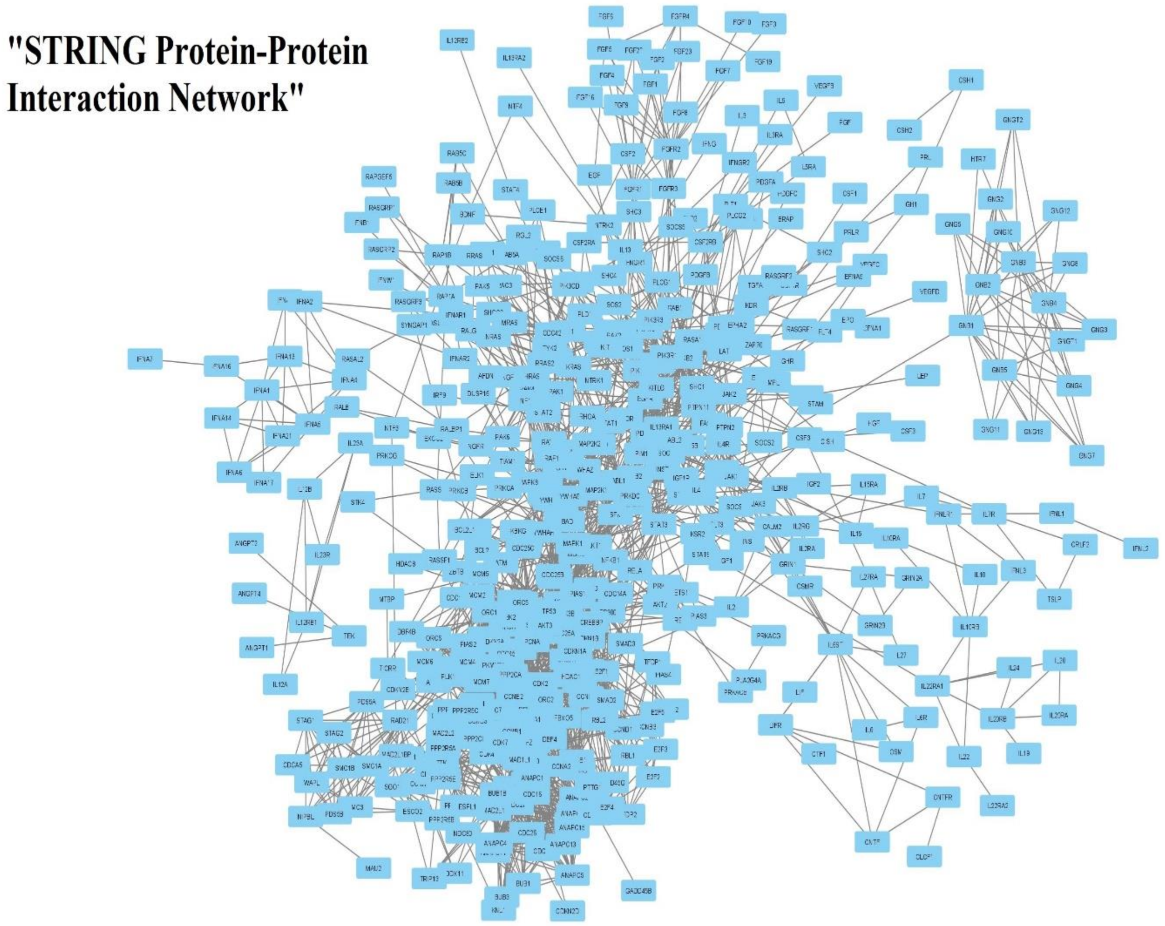

A. The gene network of the significant signaling pathways. The gens of PI3K-Akt, RAS, JAK-STAT, and cell cycle signaling pathways are used to generate the gene network.

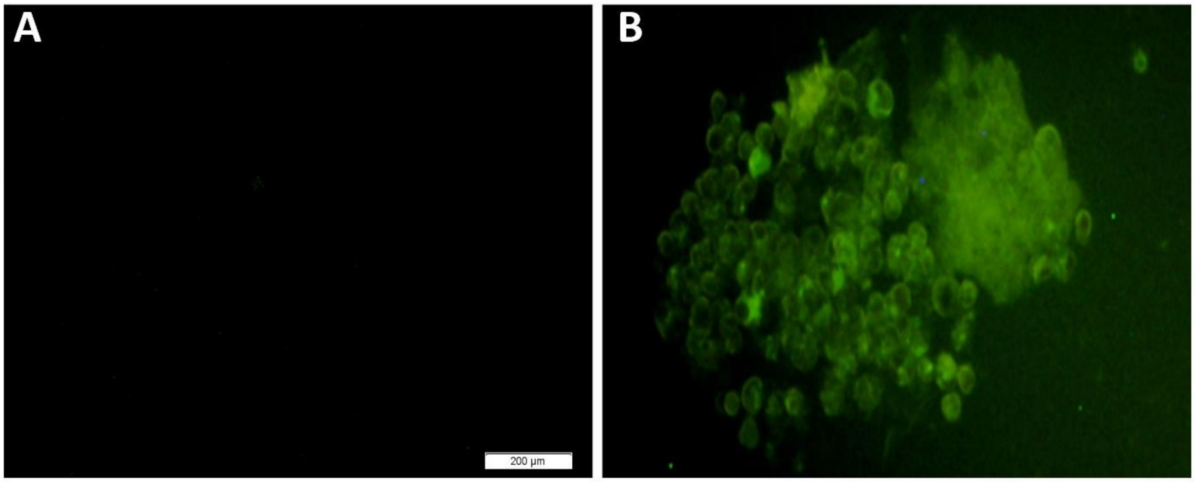

B. miRNA transfection. A, Negative control. B, HSCs transfected by FAM-labeled miRNA.

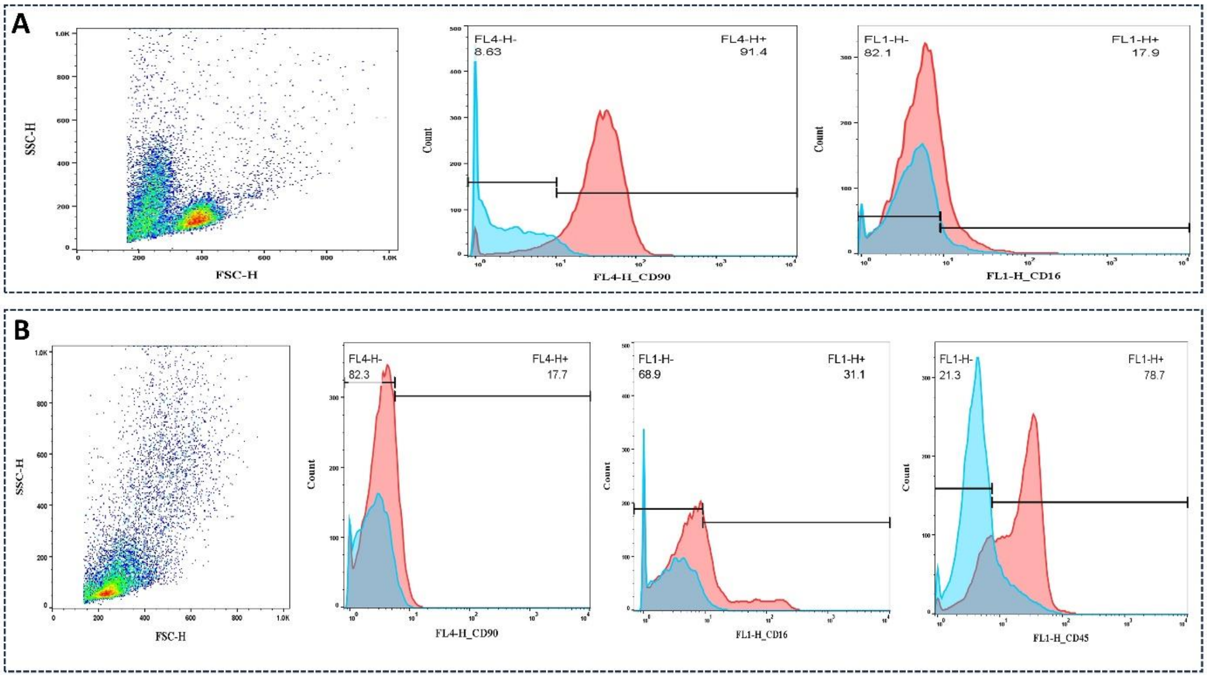

C. Flow cytometry of HSCs on the first day and the seventh day. A, The first day, the CD90 and CD16 values were estimated 94.4% and 17.9%, respectively. B, The seventh day, the CD45, CD16 and CD90 values were 78.6%, 31.1% and 17.7%, respectively.

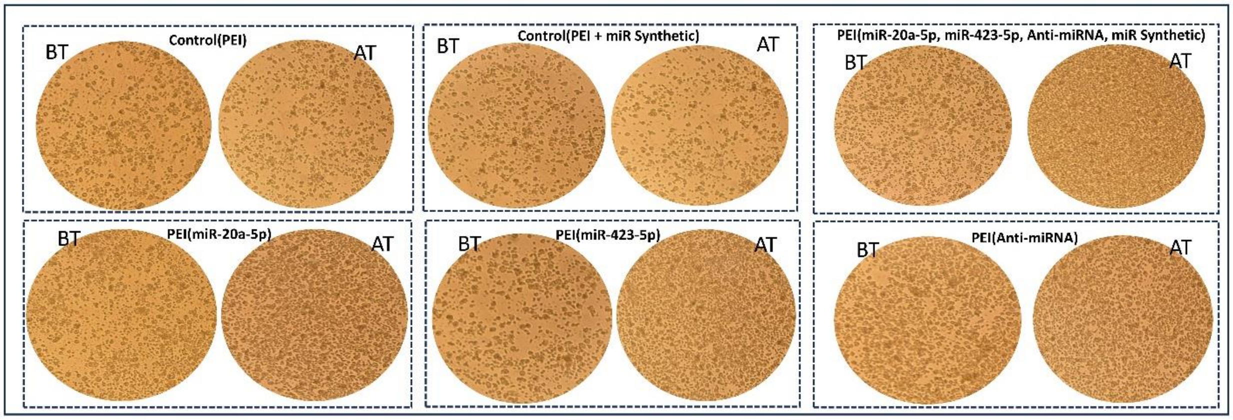

D. Microscopic images of HSCs in the study groups. Before transfection (BT). After transfection (AT).

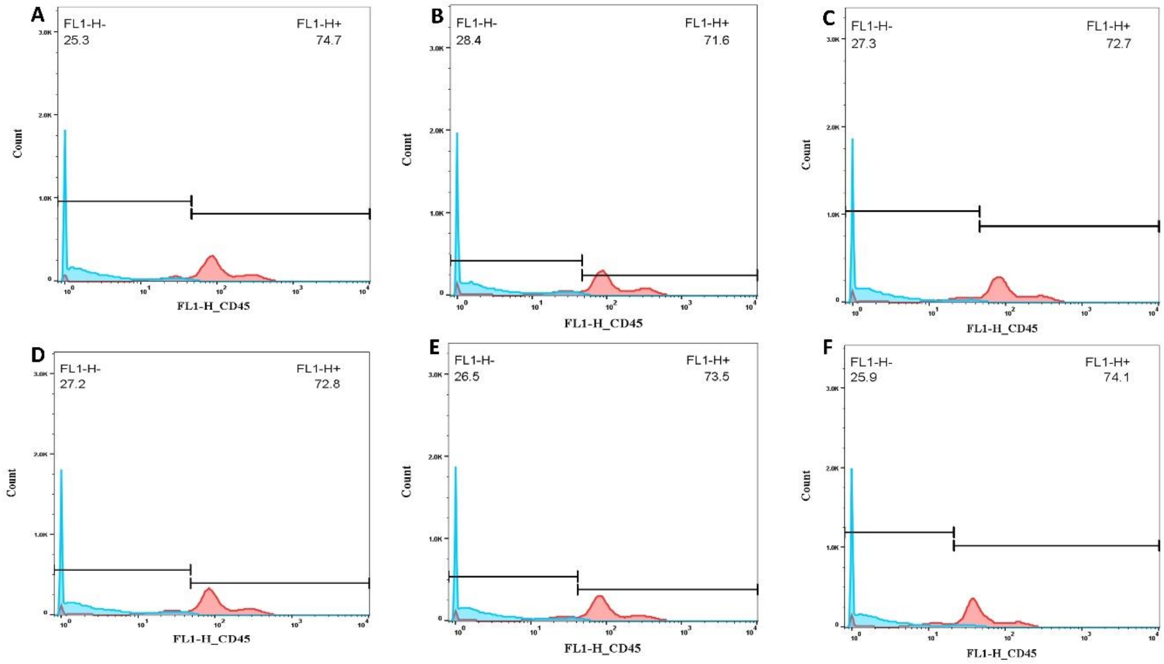

E. The CD45 values of HSCs in the study groups. A, The group treated with PEI. B, The group treated with PEI+miR-20a-5p, miR-423-5p, anti-miRNA, and miR synthetic. C, The group treated with PEI+miR-20a-5p. D, The group treated with PEI+miR-423-5p. E, The group treated with PEI+anti-miRNA. F, The group treated with PEI+miR synthetic.
